# Density Functional Theory Calculations and Infrared Spectral Analysis of Lignin

**DOI:** 10.3390/molecules29235683

**Published:** 2024-11-30

**Authors:** Zhuang Miao, Zhipeng Li, Xing Teng, Han Wang, Yingying Zhou, Yixin Qiu, Changming Li, Chunyu Liu, Yong Tan

**Affiliations:** 1 School of Physics, Changchun University of Science and Technology, Changchun 130022, China; 2022100067@mails.cust.edu.cn (Z.M.); 2023100190@mails.cust.edu.cn (Z.L.); 2024100128@mails.cust.edu.cn (H.W.); 2023100064@mails.cust.edu.cn (Y.Z.); 2023100096@mails.cust.edu.cn (Y.Q.); 2022200019@mails.cust.edu.cn (C.L.); 2 Jilin Academy of Agricultural Sciences (Northeast Agricultural Research Center of China), Changchun 130000, China; tengx450@nenu.edu.cn

**Keywords:** density functional theory, infrared spectroscopy, lignin

## Abstract

Lignin is one of the building blocks of plant cell walls, and the study of the spectral characterization of its cleavage process can help to monitor the production and reuse of straw after decay. In this paper, four theoretical model structures of lignin formed by lignin G monomers and connected by β-O-4 bonding type were optimized and calculated based on the density functional theory using the B3LYP/3-21g and B3LYP/6-311g basis sets. The results showed that the theoretical infrared spectra of lignin increased sequentially in the absorption peaks of 1500 cm^−1^ blue shift and 2932 cm^−1^ and 1200 cm^−1^ red shift, while the latter three theoretical models showed new infrared absorption peaks of 716 cm^−1^ and 823 cm^−1^ due to the presence of the β-O-4 structure, which is of great value for the theoretical spectral study of organic macromolecules and also provides data support for the spectral change in lignin in the degradation of graminaceous plants.

## 1. Introduction

**Figure 1:**
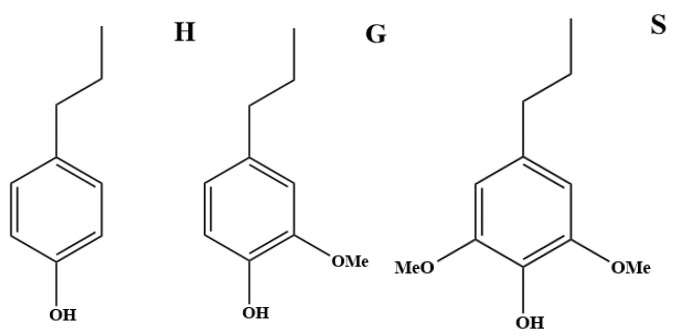
Three lignin basic units.

Lignin, as an important renewable resource, has the advantages of low cost, easy accessibility, environmental friendliness, etc., and it has a wide range of applications as a renewable, biodegradable, biocompatible, and excellent modified material matrix [1]. Ofelia Durante’s research reveals the electrical properties of lignin and develops its application in the field of memristors [2]. Lignin is essentially a class of aromatic polymers that are chemically very stable and not easily degraded [3], which mainly consists of three structural units, as shown in Figure 1, which are p-hydroxyphenyl lignin (H-type lignin), guaiacyl lignin (G-type lignin), and lilac-based lignin (S-type lignin) [4]. It has been shown that graminaceous plants contain a much larger proportion of G-type lignin than H- and S-type lignin [5]. The three phenylpropane monomers that make up lignin can form lignin macromolecular polymers through β-O-4,4-O-5, β-β, β-5, etc., [6] carbon–carbon, or carbon–oxygen bonding, of which the β-O-4 bonding type of linkage is the most important in graminaceous lignin [7]. The first all-lignin structure was proposed by Adler [8] in 1977, and lignin is considered a class of highly polymerized lignin with a variety of functional groups. A variety of functional groups and lignin is considered to be a class of high polymers with a variety of functional groups. Due to the diversity and complexity of lignin’s structure, it is very important to use efficient and accurate characterization methods to determine the structure of lignin and to understand its physical and chemical properties in order to realize the effective use of lignin.

Infrared spectroscopy, as an effective means of analyzing molecular structure, has the advantages of requiring fewer samples, easy operation, fast measurement speed [9], and the ability to analyze the molecular structure [10] and has been widely used in the fields of food [11], healthcare [12], and agriculture [13]; thus, IR spectroscopy can be used to analyze the molecular structure of lignin. Sumerskii I et al. predicted the structural properties (i.e., functionality, molar mass) of lignin by using a multivariate calibration model based on IR spectroscopy [14].

Density flood theory is a quantum mechanical method to study the electronic structure of multielectronic systems. Due to the increasing accuracy and range of action of the functionals, density functional theory (DFT) has become the most commonly used method to study the properties of molecular ground and excited states [15] and has a wide range of applications in both physics and chemistry, where it can be used to analyze the bonding modes and the structural properties of substances [16], especially to study the properties of molecules and condensed states, and is one of the most commonly used methods in the fields of condensed matter physics and computational chemistry. Chengteh Lee et al. have obtained the density functional formulas for the correlation energy and the correlation potential and have calculated them for atoms, molecules, and so on [17]. Rajabi A et al. used density functional theory to interpret the structural and spectral data, elucidate periodic trends, and predict the properties of low-valent lanthanide complexes [18]. A lot of research has been carried out at home and abroad on the theoretical calculation of excited state spectra of the organic molecules of small systems. With different methods, absorption and emission spectra can be fitted in good agreement with experimental results when the excited states of small systems are calculated. Medimagh M et al. used B3LYP/CC-PVTZ to study the effect of noncovalent interactions on the heterogeneous 4-methylbenzylammonium nitrate infrared spectral, structural, electronic, topological, and vibrational properties [19]; Dib E et al. used solid-state NMR hydrogen spectroscopy and infrared spectroscopy coupled with density functional theory calculations to reveal the mechanism of the complex hydrogen-bonded silanol network in pure silica MFI-type zeolites [20]; and Wang R confirmed the existence of close interfacial contacts between BiO_2−X_ and AgVO_3_ using DFT calculations [21]. The study by John A. Pople et al. found that the 3-21G basis set contains the same number of primitive Gaussian functions as STO-3G and should be computationally as efficient as representations for applications that require the evaluation of energy derivatives and the energy itself, and that it is less costly to apply than either the 4-21G or the 4-3 IC separate valence basis sets [22]. Julian Tirado-Rives et al. tested 622 organic compounds containing C, H, N, and O elements using the 6-31G(d), 6-31G(d, p), and 6-31+G(d, p) basis sets on the commonly used hybrid density functional B3LYP [23]. Chen Kexin combined density functional theory (DFT) and wave function analysis to reveal the pyrolysis mechanism of γ-O-4 lignin dimer model compounds, calculated free energy barriers, and reaction rates and obtained six reaction pathways [24].

In this study, using mid-infrared spectroscopy combined with density functional theory methods, we compared the differences in the infrared spectra of the four lignin structures under the base groups of B3LYP/3-21g and B3LYP/6-311g by using G-monomers and their β-O-4 bonding linkages to form G-type lignin dimers, G-type lignin trimers, and lignin oligomers, and we compared the differences in the infrared spectra of the four lignin structures under the base groups of B3LYP/3-21g and B3LYP/6-311g, and we made an evaluation on the effect of the two base groups. The results were evaluated to provide theoretical and experimental support for the return of graminaceous plants to the field.

## 2. Optimization and Calculation of Lignin Molecular Spatial Structure

**Figure 2:**
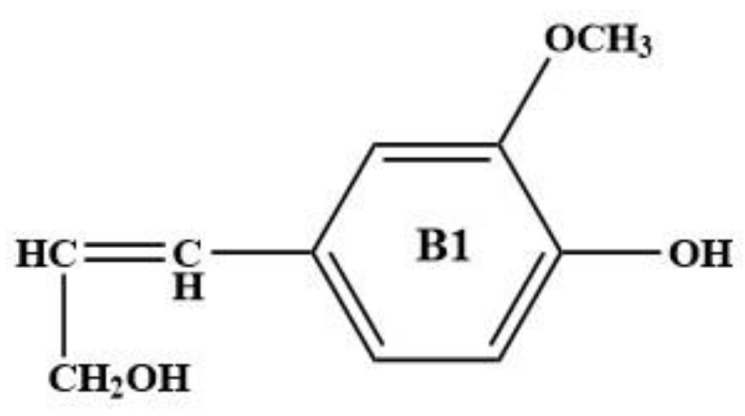
Molecular structure of lignin monomer.

**Figure 3:**
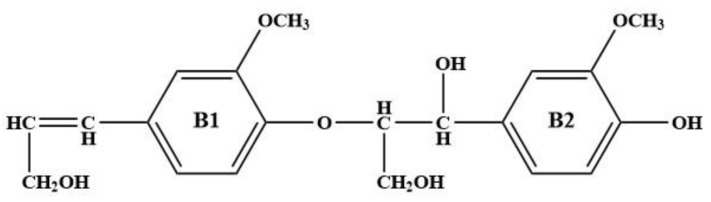
Molecular structure of lignin dimers.

**Figure 4:**
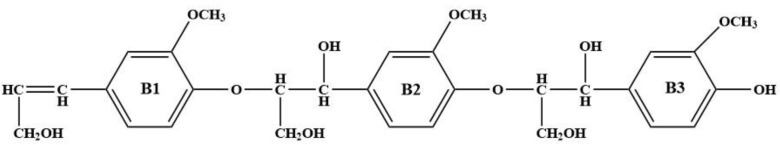
Molecular structure of lignin trimer.

**Figure 5:**
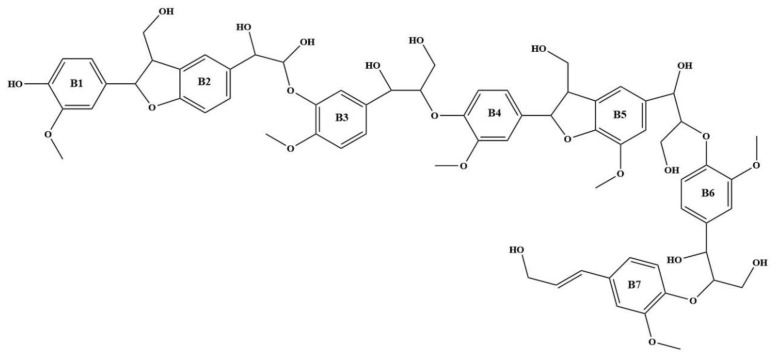
Molecular structure of lignin oligomers.

Lignin is the only naturally occurring high molecular weight polymer with an aromatic backbone, making it an exceptional source for the production of chemicals, biofuels, and materials currently obtained from fossil resources. Lignin can be obtained in large quantities from lignocellulosic residues derived from agricultural or forest biomass processing. The molecular formulae of lignin monomers, dimers, trimers, and oligomers [25] designed in this study are shown in Figure 2, Figure 3, Figure 4 and Figure 5, respectively.

**Figure 6:**
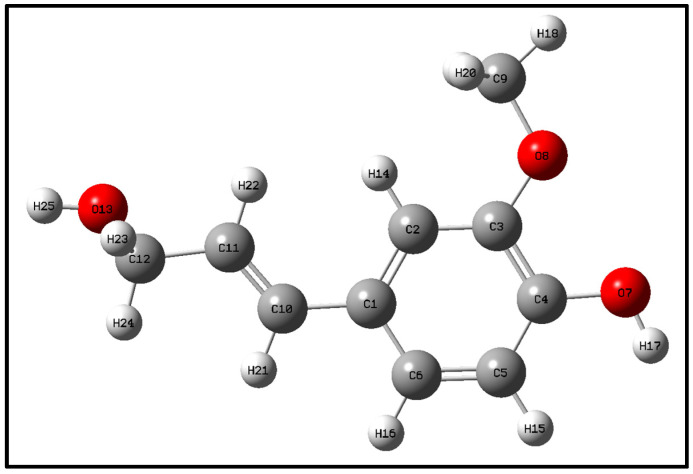
Lignin monomer space structure.

**Figure 7:**
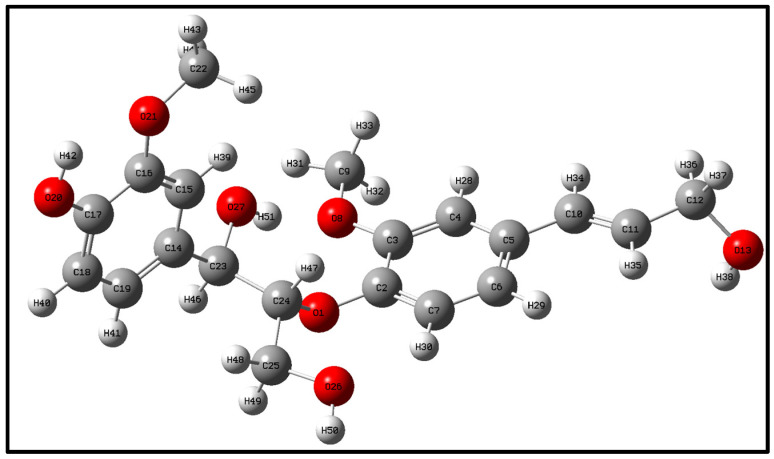
Lignin dimer space structure.

**Figure 8:**
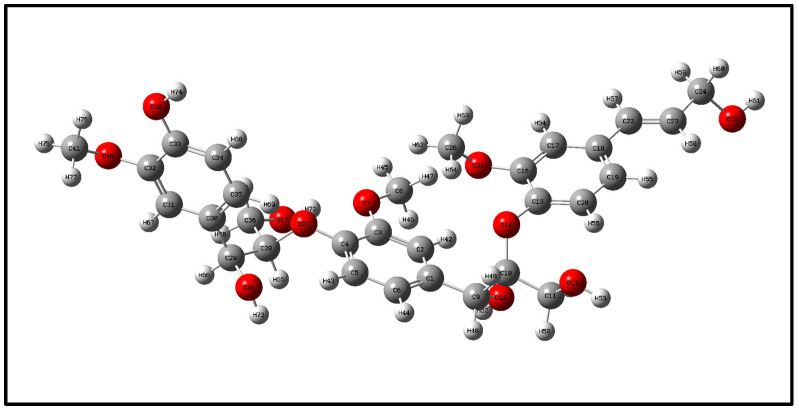
Lignin trimers space structure.

**Figure 9:**
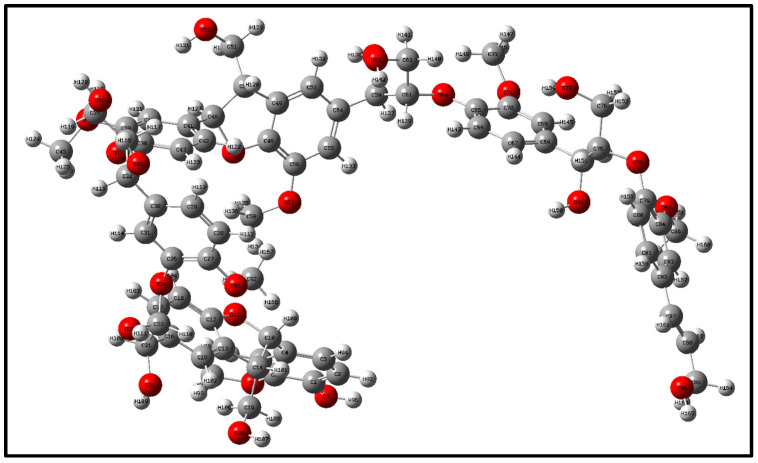
Lignin oligomer space structure.

Geometry optimization of the structures of lignin monomers, lignin dimers, lignin trimers, and lignin oligomers was carried out using the B3LYP method of density functional DFT in the base group environments of 3-21g and 6-311g. The structures of lignin monomers, lignin dimers, lignin trimers, and lignin oligomers were optimized using the B3LYP method of density functional DFT. Whether the molecular structure of lignin reaches the optimal geometrical configuration depends on its interatomic forces and the energy value of the molecule as a whole. When the interatomic force and the overall energy of the molecule reach the minimum value, the optimization is completed, and the structure is stable; the optimized lignin structure is shown in Figure 6, Figure 7, Figure 8 and Figure 9.

After the base group was coarsely optimized for the initial structure, the B3LYP\3-21g and B3LYP\6-311g base groups were selected for re-optimization, and the calculation of infrared spectra based on the optimized structures obtained the inclusion of the dispersion correction in the calculation of the spectra. The correction factors chosen for the frequency correction of the theoretical spectra were 0.965 and 0.966, respectively, and the corrections were then compared with the experimental data. The calculated results have no false frequencies, indicating that a stable structure was obtained.

## 3. Lignin Analytical Pure Experimental Spectral Acquisition

### 3.1. Instruments and Materials

The experimental equipment was a Thermo Scientific (Waltham, MA, USA) Nicolet iS50 Fourier transform infrared (FTIR) spectrometer, equipped with a He-Ne laser, with a spectral range of 400–4000 cm^−1^ and a spectral resolution better than 0.09 cm^−1^. The equipment was completely sealed in the experiment. The instrument was completely sealed, and all data collection was carried out in the laboratory environment. Lignin (CAS No. 8068-05-1) was purchased from Aladdin Reagent Co. (China/Shanghai).

### 3.2. Infrared Spectroscopy Data Acquisition

**Figure 10:**
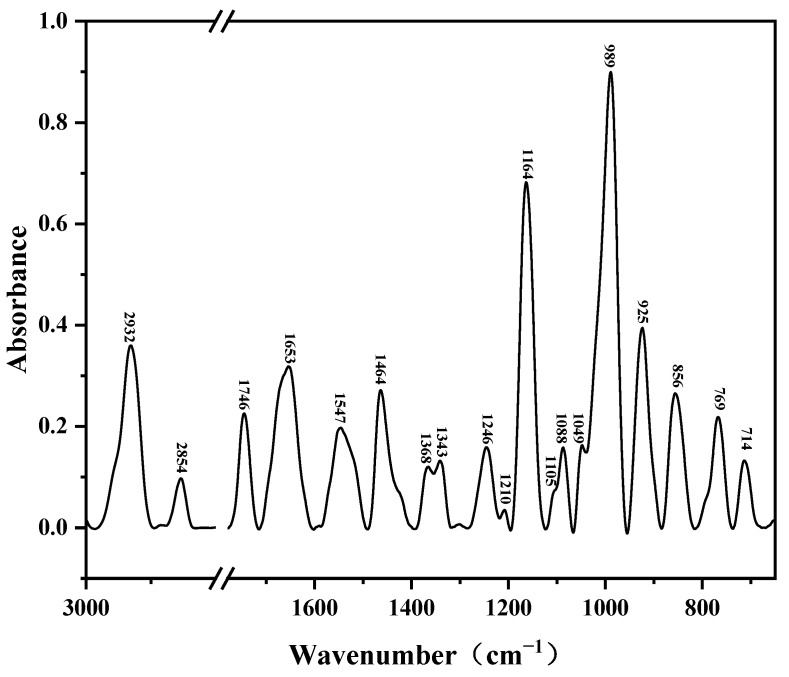
Infrared spectra of lignin after baseline treatment.

The experiment was carried out by the tablet pressing method; 1 mg of lignin and 100 mg of KBr were put into an onyx mortar, ground into a fine powder, mixed uniformly, and pressed into a semi-transparent tablet by a tablet press. The diameter of pressed lignin discs was 0.7 cm, and the thickness was 0.04 ± 0.01 mm. Then, the sample tablet was put into the sample chamber for mid-infrared spectroscopy, and the number of scans of the spectrometer was set to 32, the resolution was set to 4 cm^−1^, and the spectral detection range was set to 400–4000 cm^−1^. The original spectra were processed by removing the baseline of lignin. The raw spectra were processed to remove the baseline, and the spectra are shown in Figure 10.

## 4. Results and Discussion

### 4.1. Spectral Analysis of Lignin Molecules

After the structural optimization of the four theoretical configurations of the lignin molecule using the two basis groups of B3LYP/3-21g and B3LYP/6-311g of the density functional theory, we continued to complete the calculation of the theoretical infrared spectra of the G-monomer, dimer, trimer, and oligomer under the conditions of these two basis groups; analyzed the vibrational attribution of the various infrared peaks of the lignin from the infrared spectra; analyzed the structure of the lignin molecule in the changing structure of the lignin molecule based on the same base group optimization, calculation of the theoretical infrared spectra of G monomer, dimer, trimer, and oligomer; and analyzed the change rule of lignin’s infrared spectra when its structure becomes complex.

#### 4.1.1. Infrared Spectral Analysis of Lignin Monomer

**Figure 11:**
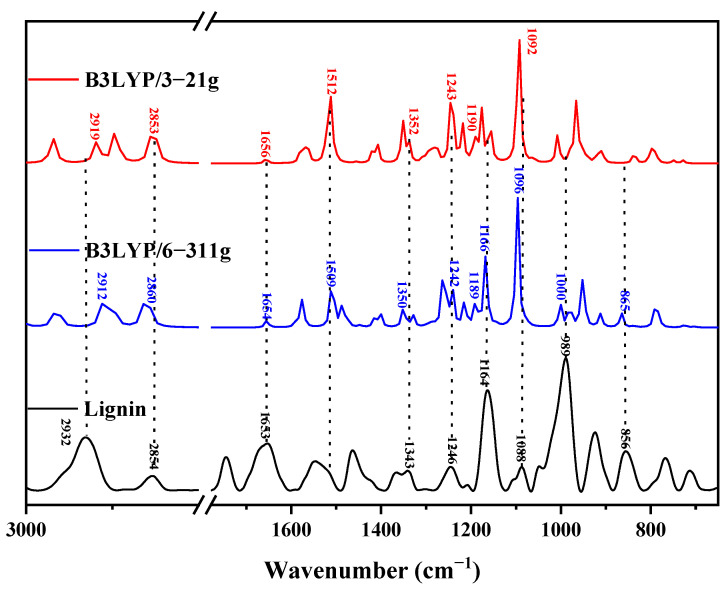
Comparison between theoretical and experimental infrared spectra of lignin monomers.

**Figure 12:**
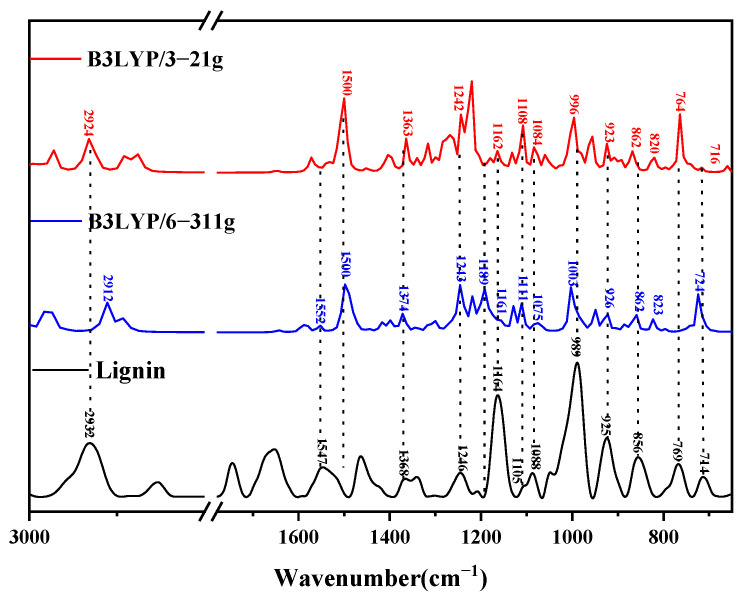
Comparison of theoretical infrared spectra and experimental spectra of lignin dimer.

**Figure 13:**
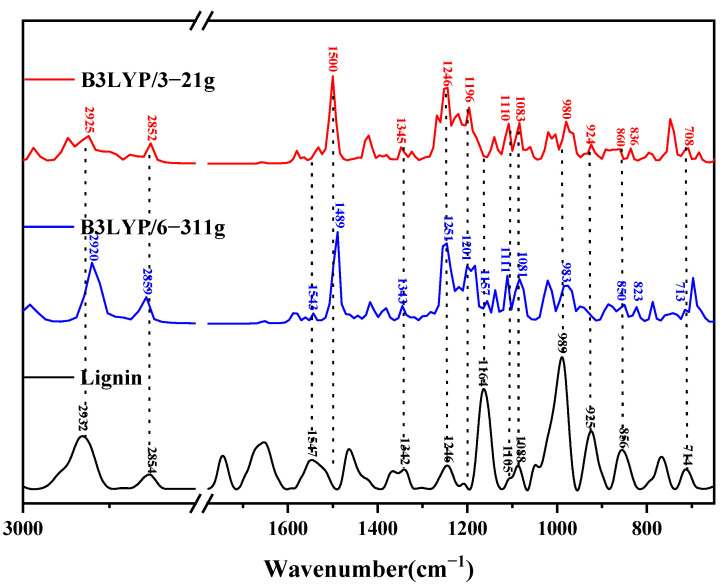
Comparison of theoretical and experimental infrared spectra of lignin trimers.

**Figure 14:**
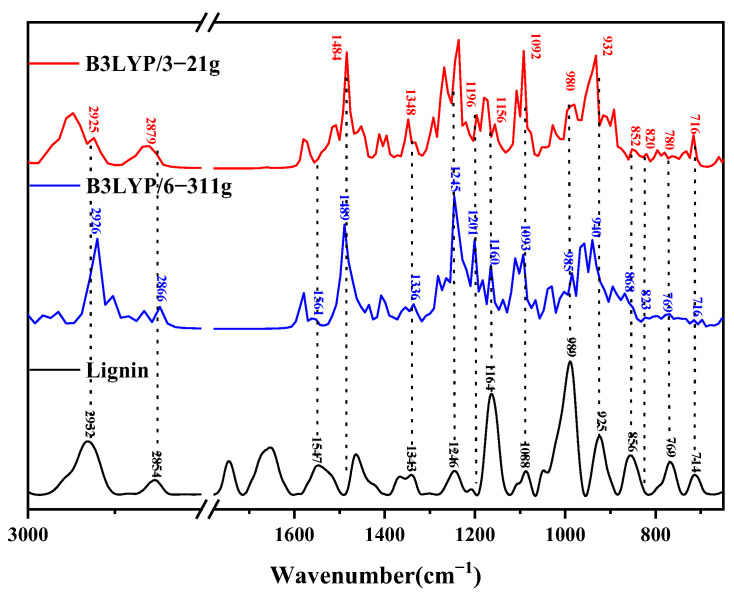
Comparison of theoretical and experimental infrared spectra of lignin oligomers.

Using the optimized lignin monomer, dimer, trimer, and oligomer molecular model calculation to obtain the theoretical infrared spectra, the experimentally measured lignin is shown in Figure 11, Figure 12, Figure 13 and Figure 14, respectively. Table 1, Table 2, Table 3 and Table 4 list the molecular vibrations corresponding to each of its peak positions in the theoretical spectra of lignin under the conditions of the two different computational basis sets; the peak at position 2912 cm^−1^ originates from the asymmetric stretching vibration of C12-H23H24; whereas, the absorption peak at 2854 cm^−1^ is likewise caused by the asymmetric stretching of C12-H23H24; the absorption peak near 1653 cm^−1^ is attributed to the stretching vibration of C10=C11; the absorption peak at 1509 cm^−1^ is caused by the deformation vibration of the benzene ring B1 together with the stretching vibration of C4-O7; the absorption peak at 1343 cm^−1^ is derived from the deformation vibration of the benzene ring B1 as well as the swaying vibration of O7-H17 and C10-H21; the absorption peak at 1246 cm^−1^ is caused by the deformation of the benzene ring, the asymmetric stretching vibration of C3-O8-C9, the asymmetric stretching vibration of C3-O8-C9, and the stretching vibration of C1-C10; the absorption peak at 1190 cm^−1^ reflects the combined effect of the rocking vibration of C9-H18H19H20 and the deformation vibration of the benzene ring B1; and the absorption peak at 1164 cm^−1^ originates from the deformation vibration of the benzene ring B1, the out-of-plane rocking vibration of C9-H19H20, and the torsional vibration of C9-H18H19. The absorption peak at 1088 cm^−1^ is caused by the rocking vibration of O7-H17 and the deformation vibration of benzene ring B1; the absorption peak at 989 cm^−1^ originates from the deformation of benzene ring B1 as well as the telescopic vibration of C9-O8; and the absorption peak at the position of 855 cm^−1^ reflects the result of the combined effect of the deformation of the benzene ring and the rocking vibration of C12-H23H24 [7].

#### 4.1.2. Infrared Spectral Analysis of Lignin Dimers

The theoretical infrared spectra were calculated using the optimized lignin dimer molecular model, and the comparison with experimentally measured lignin is shown in Figure 12. The theoretical IR spectra of the dimer molecule were under two different, optimized, calculated basis sets, where the horizontal coordinate is the wave number, and the vertical coordinate is the IR signal intensity. Table 2 lists the molecular vibrations corresponding to each peak position in the theoretical spectra of lignin under the conditions of the two different computational basis groups. The peak at 2932 cm^−1^ originates from the symmetric telescopic vibration of C25-H48H49; the absorption peak near 1547 cm^−1^ is due to the deformation vibration of the benzene ring B1 in conjunction with the telescopic vibration of C10-C11; near 1500 cm^−1^, the peak appearance is the result of the deformation vibration of benzene rings B1 and B2 and the rocking vibration of C22-H44H45; the peak at 1368 cm^−1^ is generated by the out-of-plane rocking vibration of C12-H36H37 and the rocking vibration of O13-H38; the absorption peak at 1246 cm^−1^ is the combined effect of the deformation of benzene ring B2, the rocking vibration of C11-H35, and the torsional vibration of C25-H48H49; the absorption peak near 1190 cm^−1^ is a manifestation of these combined. The peak at 1190 cm^−1^ is caused by the deformation vibration of benzene ring B2 and the swaying vibration of C22-H43H44H45 and O20-H42; the peak at 1164 cm^−1^ originates from the deformation of benzene ring B2 and the twisting vibration of C12-H36H37; the peak at 1105 cm^−1^ is the result of the deformation vibration of benzene ring B2 and the swaying vibration of O28-H42; the peak at 1088 cm^−1^ is the result of the deformation of benzene ring B2 and the swaying vibration of O28-H42; the peak at 1088 cm^−1^ is the result of the deformation of benzene ring B2 and the swaying vibration of C12-H48H49, as a result of the deformation of benzene ring B2; the absorption peak at 1088 cm^−1^ is related to the telescopic vibration of C11-C12 and the rocking vibration of O13-H38; the peak at 989 cm^−1^ is a result of the combination of the telescopic vibration of O21-C22 and the deformation vibration of benzene ring B2; the absorption peak at 856 cm^−1^ reflects the deformation of benzene ring B1; whereas, the absorption peak at 823 cm^−1^ is a result of the C14=C19-H41 and C17=C18-H40; the peak at 769 cm^−1^ originates from the rocking vibration of O27-H51; and finally, the peak at 713 cm^−1^ is due to the combination of the deformation of benzene ring B2 and the rocking vibration of O27-H51.

#### 4.1.3. Infrared Spectral Analysis of Lignin Trimer

The theoretical infrared spectra were calculated using the optimized molecular model of the lignin trimer, and the comparison with the experimentally measured lignin is shown in Figure 13. Table 3 lists the molecular vibrations corresponding to each of its peak positions in the theoretical spectra of lignin under two different computational basis group conditions. The peak at 2932 cm^−1^ originates from the C-H telescoping vibration of the methyl group (C37-H72H73H74), where C37-H73H74 is a symmetric telescoping vibration; whereas, the peak at 2854 cm^−1^ is related to the rocking vibration of C10-H50. The absorption peak near 1547 cm^−1^, on the other hand, originates from the deformation vibration of the benzene ring B3 and the C-H out-of-plane rocking vibration on the methyl group (C26-H62H63H64); the peak at 1490 cm^−1^ reflects the rocking vibration of C26-H62H63H64; the absorption peak at 1342 cm^−1^ is from the C9-H48, C10-H49, and O12-H52 rocking vibrations; the peak at 1246 cm^−1^ is caused by the breathing vibration of benzene ring B1; the absorption peak at 1201 cm^−1^ is caused by the combination of the deformation vibration of benzene ring B1 and the rocking vibration of O13-H53; the peak at position 1164 cm^−1^ contains the outward torsion of the C-H face on the methyl group (C37-H72H73H74), the torsion of C40-H76H77, and the rocking vibration of O35-H71; the peak at 1105 cm^−1^ corresponds to the deformation vibration of benzene ring B1; the peak at position 1088 cm^−1^ reflects the deformation of benzene ring B1 and the rocking vibration of O39-H74; the peak at 989 cm^−1^ originates from the stretching vibration of C36-O37; the peak at position 925 cm^−1^ is the stretching vibration of O7-H8 with C2-H42 and C17-H54; the absorption peak near 856 cm^−1^ reflects the deformation of benzene rings B1 and B2; the absorption peak at position 823 cm^−1^ is caused by the rocking vibrations of C22-H57 and C24-H59H60; and the peak at 714 cm^−1^ is due to the deformation vibrations of benzene rings B1, B2, and B3.

#### 4.1.4. Infrared Spectral Analysis of Lignin Oligomers

The absorption peak at 2932 cm^−1^ originates from the asymmetric stretching vibration of C35-H118H119 (Table 4); the peak at the position of 2854 cm^−1^ is attributed to the asymmetric stretching vibration of C86-H159H160; the peak near 1547 cm^−1^ is triggered by the deformation vibration of the benzene ring B4; the absorption peak near 1489 cm^−1^ reflects the C75-H152H153 rocking vibration; the absorption peak at position 1343 cm^−1^ is associated with the rocking vibration of C21-H100 and O20-H107 and the torsional vibration of C19-H105H106; the absorption peak at position 1246 cm^−1^ originates from the respiratory vibration of benzene ring B4 and the rocking vibration of C50-H128; the absorption peak at position 1201 cm^−1^ is a result of benzene ring B7’s deformation vibration and the stretching vibration of C79-O78; the peak at 1164 cm^−1^ is related to the out-of-plane rocking vibration of C58-H135H134H136; the absorption peak at 1088 cm^−1^ is related to the rocking vibration of O7-H96 and the deformation vibration of benzene ring B1; the peak at 989 cm^−1^ comes from the stretching vibration of O71-C72 and the benzene ring B6; the absorption peak at 925 cm^−1^ is the result of the combined action of the telescopic vibration of C10-O11 and the deformation vibration of benzene ring B5; the peak at 856 cm^−1^ corresponds to the swaying vibration of C40-H121 and the telescopic vibration of C46-O47; the absorption peak at 823 cm^−1^ is caused by the swaying vibration of C17-H103 and C18-H104; the peak at 769 cm^−1^ reflects the deformation vibrations of benzene rings B1 and B2; while the peak at 713 cm^−1^ is associated with the rocking vibration of O33-H116 (see Figure 14).

According to the above study, we can find that the lignin molecular vibration is attributed mainly to the carbon–hydrogen bond wobbles, stretching vibration benzene ring deformation vibration, and so on. For example, in the infrared peak at 2924 cm^−1^, the theoretical spectra of the four lignin structures have corresponding peaks here, and they are all due to the symmetric telescopic vibration of the C-H bond, except that the position of the vibrating functional group is not the same; the monomer and trimer are the C-H telescopic vibration of the methyl group; and the dimer and the oligomer are due to the C-H telescopic vibration of the other parts of the molecule.

Under different base group optimization and calculation conditions, the theoretical spectra obtained are not exactly the same, and some peaks will be shifted or disappeared, which may be caused by the influence of optimization factors. By comparing the magnitude of the shifts in the horizontal coordinates of the peaks and the number of peaks in the infrared spectra of the four lignin molecular models under the optimization and calculation of the two basis groups, it is found that the effect of the calculation of the B3LYP/6-311g basis group is significantly better than that of the B3LYP/3-21g.

### 4.2. Comparative Analysis of Infrared Spectra of Lignin Molecules with Different Structures

In order to explore the difference between the mid-infrared spectra of lignin long chains and short chains, according to the previous results, the theoretical spectra of lignin molecules calculated by the B3LYP/6-311g group were used as the research target, and it was found that, from lignin oligomers to lignin monomers, as the structure of lignin becomes simpler, the intensities of most of the peaks are obviously weakened, and the number of peaks shows a decreasing trend, which may be due to the fact that the number and types of internal functional groups are also reduced, which makes the types and intensities of the functional group vibrations also decrease accordingly. This may be due to the fact that when the molecular structure becomes simpler, the number and types of internal functional groups are also reduced, resulting in a corresponding weakening of functional group vibration types and intensities.

**Figure 15:**
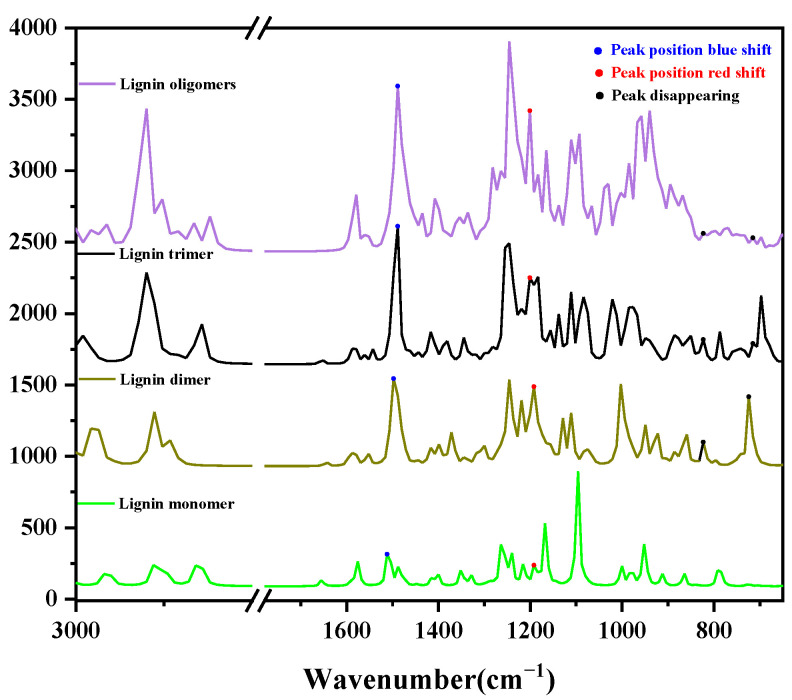
Comparison of infrared spectra of lignin molecules with different structures calculated by the optimization of B3LYP/6-311g.

**Figure 16:**
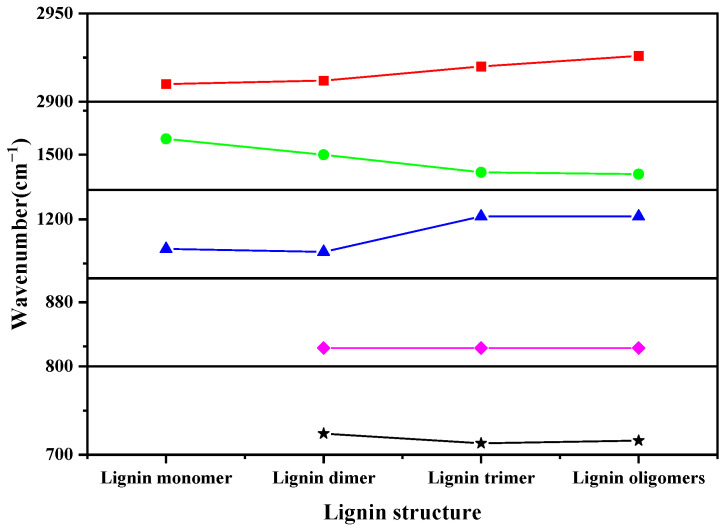
Comparison of peak distributions of theoretically calculated infrared spectra of lignin. (Figure 16 is a supplementary explanation of Figure 15, from which we can more intuitively see the variation pattern of peak positions).

In addition to changes in peak intensity, we can also find that the location of the peaks in the infrared spectra of lignin monomer, lignin dimer, lignin trimer, and lignin oligomer is not exactly the same, as shown in Figure 15. Its peak distribution pattern is shown in Figure 16. For example, at about 2920 cm^−1^, monomer, dimer, trimer, and oligomer here are corresponding to the infrared absorption peaks and red shift phenomenon, from the point of view of vibrational attribution. The infrared absorption peaks of dimer and trimer here are due to the symmetric stretching vibration of C-H on the methyl group, while the vibrational attribution of the infrared absorption peaks of monomer and oligomer here is due to the anti-symmetric stretching vibration of C-H, and the C and H molecules are not located on the methyl group; near 1500 cm^−1^, the peak position of the absorption peaks appears to have a blue-shifted phenomenon, as the structure of the lignin becomes simpler. The vibration of the lignin monomer here is caused by the shear rocking vibration of C9-H19H20 and the torsion of C9-H18H19; the peak of the lignin dimer here is caused by the torsion vibration of C9-H32H33; the peak of lignin trimer here is caused by the deformation of the benzene ring B3 and the deformation of the C-H surface on the methyl group (C23-H59H60H61) and the C-H surface on the methyl group (C37-H72H73H74); and the C and H molecules are not located on the methyl group. On the C-H out-of-plane rocking vibration, the peak of lignin oligomer here is due to the O33-H116 rocking vibration and C35-H118H119 shear rocking vibration; while the peak position of the absorption peak at about 1200 cm^−1^ shows a redshift phenomenon, the lignin monomer here is caused by the torsional vibration of the vibration C12-H23H24; the lignin dimer here peak is caused by the deformation of benzene ring B1 and the rocking vibration of O27-H51 and C23-H46; the peak of lignin trimer here is caused by the deformation of benzene ring B1 and the telescopic vibration of C18-O21; and the peak of lignin oligomer here is due to the deformation of benzene ring B7 and the rocking vibration of O74-H150, which is not a good explanation of the change in the peak position in terms of the vibrational attribution; at 823 cm^−1^, we can find that, in addition to monomers, oligomers, trimers, and dimers have infrared peaks here; the peaks of oligomers here are due to the deformation of benzene rings B1 and B2; the peaks of trimers here are due to the deformation of benzene ring B3 and the twisting of C40-H77H78; and the peaks of dimers here are due to the deformation of benzene ring B2, which can be analyzed as the reason for the change in peak positions. The functional groups in the molecular structure of dimers, trimers, and oligomers all contain the functional group types in the monomers, but the β-O-4 bond structure is missing in the monomers, so the spectral deviation here may be due to the absence of the β-O-4 structure in the monomers; the absence of peaks in the theoretical infrared spectra of lignin-only monomers at 716 cm^−1^ also occurs. Combined with the vibrational attribution made earlier, the vibrational attribution of lignin dimers, trimers, and oligomers at this location includes the O-H vibration, and the position of this O-H bond is located in the connection between the two structural units; obviously, the monomer does not exist in this structure, which also explains the reason for the absence of absorption peaks at this location in the monomer.

## 5. Conclusions

In order to find the change rule of lignin cleavage inside the plant when it is decaying and to determine the specific degree of lignin cleavage, this thesis adopted a density functional theory combined with infrared spectroscopy to simplify the molecular structure of lignin, starting from the G monomer, and assuming that it was connected to form a multilayered molecule in the form of β-O-4. Then, these ideal lignin molecular structures were optimized, and an analytical model was established using B3LYP/3-21g and B3LYP/6-311g, which were the two base groups. They were optimized, the analytical models were built to calculate their infrared spectra, and the vibrational modes of the absorption peaks of each spectrum were determined to be attributed to them, and meanwhile, after comparison, it was found that the calculation of B3LYP/6-311g was better than that of B3LYP/3-21g, and the theoretical infrared spectra of the lignin by comparing the oligomers, trimers, dimers, and monomers were found. The difference between the IR spectra of macromolecules and small molecules was found, and an explanation was given from the molecular vibration point of view, which provided data support for the theoretical study of lignin and a reference for the detection of lignin cleavage in gramineous plants by spectroscopic means.

## Figures and Tables

**Table 1:** Vibrational attribution of lignin monomers.

Experiment (cm^−1^)	Theory (cm^−1^)		Vibration
	B3LYP/3-21g	B3LYP/6-311g	
2932 (S)	2918 (m)	2910 (m)	ν (C12-H23H24)
2854 (w)	2853 (S)	2860 (S)	ν (C12-H23H24)
	1512 (S)	1509 (m)	δ (B1) ν (C4-O7)
1653 (S)	1656 (w)	1654 (w)	ν (C10=C11)
1343 (w)	1352 (m)	1350 (m)	δ (B1) ρ (O7-H17) ρ (C10-H21)
1246 (w)	1243 (S)	1242 (S)	δ (B1) νs (C3-O8-C9) ν (C1-C10)
	1189 (w)	1190 (w)	δ (B1) ρ (C9-H18H19H20)
1164 (S)	1158 (m)	1166 (S)	δ (B1) ρ (C9-H19H20) τ (C9-H18H19)
1088 (w)	1092 (w)	1096 (S)	ρ (O7-H17) δ (B1)
989 (S)		1002 (m)	ν (C9-O8) δ (B1)
856 (m)		865 (w)	δ (B1) ρ (C12-H23H24)

Calculated numbers at B3LYP/3-21g, B3LYP/6-311g basis sets of theory. S, strong; m, medium; w, weak; δ, deformation; ρ, rocking; τ, torsion; ν, stretching; s, symmetric.

**Table 2:** Vibration attribution of lignin dimer.

Experiment (cm^−1^)	Theory (cm^−1^)		Vibration
	B3LYP/3-21g	B3LYP/6-311g	
2932 (S)	2924 (S)	2912 (S)	νa (C25-H48H49)
1547 (w)		1552 (S)	δ (B1) ν (C10=C11)
	1500 (S)	1500 (S)	δ (B1, B2) ρ (C22-H44H45)
1368 (w)	1363 (S)	1374 (m)	ρ (C12-H36H37) ρ (O13-H38)
1246 (w)	1242 (S)	1243 (S)	δ (B2) ρ (C11-H35) ρ (C25-H48H49)
		1189 (m)	δ (B2) ρ (C22-H43H44H45) ρ (O20-H42)
1164 (S)	1162 (m)	1161 (w)	ρ (C9-H31H33) τ (C12-H36H37) δ (B2)
1105 (w)	1108 (m)	1111 (w)	δ (B2) ρ (O28-H42)
1088 (m)	1084 (w)	1075 (w)	ν (C11-C12) ρ (O13-H38)
989 (S)	996 (S)	1003 (S)	δ (B2) ν (O21-C22)
925 (w)	923 (m)	926 (m)	ν (O13-C12) ρ (C10=C11-H35)
856 (m)	862 (m)	862 (m)	δ (B1)
	820 (w)	823 (w)	ρ (C14=C19-H41) ρ (C17=C18-H40)
769 (m)	764 (S)		ρ (O27-H51)
714 (m)	716 (w)	724 (S)	ρ (O27-H51) δ (B2)

Calculated numbers at B3LYP/3-21g, B3LYP/6-311g basis sets of theory. S, strong; m, medium; w, weak; δ, deformation; ρ, rocking; τ, torsion; a, asymmetric; ν, stretching.

**Table 3:** Vibrational attribution of lignin trimers.

Experiment (cm^−1^)	Theory (cm^−1^)		Vibration
	B3LYP/3-21g	B3LYP/6-311g	
2932 (m)	2925 (m)	2920 (m)	νs (C37-H73H74) ν (C37-H72)
2854 (w)	2852 (w)	2859 (w)	ρ (C10-H50)
1547 (w)		1543 (S)	δ (B3) ρ (C26-H62H63H64)
		1490 (S)	ρ (C26-H62H63H64)
1342 (w)	1345 (w)	1343 (w)	ρ (C9-H48) ρ (C10-H49) ρ (O12-H52)
1246 (w)	1246 (S)	1251 (m)	δ (B1)
	1196 (m)	1201 (w)	δ (B1) ρ (O13-H53)
1164 (S)		1157 (w)	τ (C37-H72H73H74) τ (C40-H76H77) ρ (O35-H71)
1105 (w)	1110 (m)	1111 (m)	δ (B1)
1088 (m)	1083 (m)	1081 (m)	δ (B1) ρ (O39-H74)
989 (S)	980 (m)	983 (w)	ν (C36-O37)
925 (S)	924 (w)		ν (O7-H8) ρ (C2-H42) ρ (C17-H54)
856 (w)	860 (w)	850 (w)	δ (B1, B2)
	836 (m)	823 (m)	ρ (C22-H57) ρ (C24-H59H60)
714 (w)	708 (m)	713 (m)	δ (B1, B2, B3) ρ (O27-H66)

Calculated numbers at B3LYP/3-21g, B3LYP/6-311g basis sets of theory. S, strong; m, medium; w, weak; δ, deformation; ρ, rocking; τ, torsion; s, symmetric; ν, stretching.

**Table 4:** Vibrational attribution of lignin oligomers.

Experiment (cm^−1^)	Theory (cm^−1^)		Vibration
	B3LYP/3-21g	B3LYP/6-311g	
2932 (m)	2925 (m)	2926 (S)	ν (C37-H73H74)
2854 (w)		2866 (w)	νa (C86-H159H160)
1547 (m)		1561 (w)	δ (B4)
	1484 (S)	1489 (S)	ρ (C75-H152H153)
1343 (w)	1348 (m)	1336 (w)	ρ (C21-H100) τ (C19-H105H106) ρ (O20-H107)
1246 (w)		1245 (S)	δ (B4) ρ (C50-H128)
	1196 (m)	1201 (S)	δ (B7) ν (C79-O78)
1164 (s)	1156 (m)	1160 (m)	ρ (C58-H135H134H136)
1088 (m)	1092 (S)	1093 (m)	ρ (O7-H96) δ (B1)
989 (S)	980 (w)	985 (w)	δ (B6) ν (O71-C72)
925 (m)	932 (S)	940 (S)	ν (C10-O11) δ (B5)
856 (w)	852 (w)	868 (m)	ρ (C40-H121) ν (C46-O47)
	820 (w)	823 (w)	ρ (C17-H103) ρ (C18-H104)
769 (w)	780 (w)	769 (w)	δ (B1) δ (B2)
714 (w)	716 (m)	716 (w)	ρ (O33-H116)

Calculated numbers at B3LYP/3-21g, B3LYP/6-311g basis sets of theory. S, strong; m, medium; w, weak; δ, deformation; ρ, rocking; τ, torsion; s, symmetric; a, asymmetric; ν, stretching.

## Data Availability

The data presented in this study are available in this paper.
